# Endoscopic posterior resection of C2 osteoid osteoma with 3 years follow-up

**DOI:** 10.1016/j.bas.2026.106007

**Published:** 2026-03-21

**Authors:** Cristian CorreaV, Diego ContrerasS, Matias NahuelpanS, Camila SalazarC, Facundo Van Isseldyk, Jin- Sung Kim

**Affiliations:** a Spine Center, Clinica Las Condes, Santiago, Chile; b Department of Orthopaedics and Traumatology, Padre Las Casas, Chile; c Department of Orthopaedics and Traumatology, Hospital Hernán Henriquez Aravena, Temuco, Chile; d Department of Neurosurgery, Hospital Privado de Rosario, Rosario, Argentina; e University of Korea, 222 Banpo-daero, Seocho-Gu, Seoul, 06591, South Korea

**Keywords:** C2 osteoid osteoma, Spinal endoscopy, Endoscopic decompression

## Abstract

**Study design:**

Case report and literature review.

**Objective:**

To present and describe the surgical technique of the case with 3 years follow-up, the longest one reported to date involving posterior endoscopic management of a C2 osteoid osteoma.

**Background:**

Summary: The development of spinal endoscopic techniques has enabled the treatment of degenerative spinal disorders and has expanded their indications to include spinal tumor pathology. This report represents the case with the longest follow-up reported to date regarding the use of endoscopic techniques in the treatment of cervical osteoid osteoma.

**Methods:**

A 10-year-old patient presented with a 2-year history of cervical pain. Diagnostic imaging with Computed Tomography (CT) and Magnetic Resonance (MR) revealed an osteoid osteoma located in the C2 lamina and pedicle. The patient underwent posterior cervical endoscopy with complete tumor excision.

**Results:**

Clinical and radiological follow-up demonstrated tumor resection with a significant reduction of symptoms. No signs of tumor recurrence were observed at 3 years of follow-up.

**Conclusion:**

Endoscopic techniques have expanded their indications to include spinal tumor pathology. In experienced hands, this is a safe technique that allows direct visualization of tumor resection.

Its advantages include minimal morbidity, reduced postoperative pain and discomfort, decreased analgesic dependence, and improved cosmetic outcomes.

## Highlights

• Pediatic patient.
• Full endoscopic posterior resection is feasible for osteoid osteoma in upper cervical spine.
• Direct endoscopic visualization allows tumor resection in contact with the spinal cord.
• Ultra–minimally invasive approach avoids open surgery and potential fixation-related morbidity used in cases Osteoid Osteoma near to spinal cord.

## 1 Introduction

Osteoid osteoma (OO) is a benign bone tumor that most commonly affects the long bones of young patients (Boriani et al., 1997).

Spinal involvement occurs in approximately 20% of cases, most frequently affecting the posterior elements of the thoracolumbar vertebrae and only rarely the cervical spine. Histologically, osteoid osteomas are characterized by the presence of active osteoblasts producing immature bone tissue, along with increased local prostaglandin synthesis, which constitutes the main pathophysiological mechanism responsible for the typical clinical presentation of localized nocturnal pain (Bonnevialle and Railhac, 2001).

Initial treatment of choice is conservative, using Nonsteroidal Anti-inflammatory Drugs (NSAIDs) for symptomatic relief. Surgical treatment is reserved for refractory cases.

Conventional open resection requires extensive dissection of the paravertebral musculature and disruption of the facet joints to achieve complete nidus excision. This may compromise spinal stability and is associated with greater blood loss, longer operative time, and prolonged hospitalization.

Although spontaneous regression has been described, the duration of this process is unpredictable and may require prolonged symptomatic treatment. In our case, after 2 years of refractory symptoms despite NSAID therapy, surgical treatment was indicated.

Minimally invasive alternatives include radiofrequency ablation and tubular resection techniques, which have demonstrated favorable outcomes in the literature; however, they carry significant risks when the lesion is located adjacent to neural or vascular structures (Samaha et al., 2005).

We present the case with the longest clinical and radiological follow-up reported to date of posterior endoscopic treatment of a C2 osteoid osteoma, with a 3-year follow-up.

## 2 Materials and methods

A 10-year-old male patient presented with a 2-year history of severe nocturnal cervical pain, rated 10/10 on the Visual Analog Scale (VAS). Physical examination revealed limited cervical rotation to the left, without neurological deficits.

**Fig. 1:**
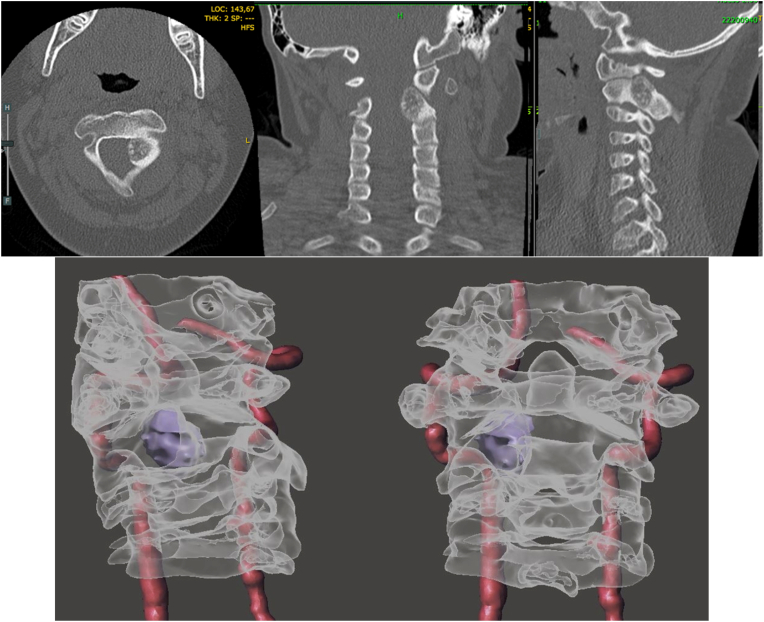
a) Axial, coronal, and sagittal CT bone window images showing a tumoral lesion in the left lamina of C2. b) Three-dimensional angio-CT reconstruction demonstrating the close relationship between the tumor and vascular structures.

CT demonstrated a lytic lesion with sclerotic margins measuring 18 × 14 mm in the left lamina of C2, involving the ipsilateral pedicle and extending to the C1–C2 joint, located ventral to the venous plexus of the second cervical root, consistent with osteoid osteoma (Fig. 1).

MR revealed a focal lesion with STIR hyperintensity causing deformation of the dural sac in contact with the spinal cord, without signs of myelopathy.

Percutaneous radiofrequency ablation was ruled out due to the high risk associated with the lesion's proximity to critical neural and vascular structures. Therefore, surgical treatment was chosen, using the endoscopic approach as separation surgery and intralesional curettage strategy combined with radiofrequency ablation under direct visualization as well. This was performed through a posterior interlaminar approach guided by fluoroscopy and continuous neuromonitoring.

## 3 Surgical technique description

Under general anesthesia, the patient was positioned prone on a radiolucent table using a Mayfield head holder. Surgery was performed under continuous neuromonitoring. After skin preparation, antisepsis, and sterile draping, the approach was initiated.

Level localization was first performed using fluoroscopic guidance. A posterior lateral approach was then carried out at the level of the left C2 pedicle. A camera working cannula from the RIWOspine® system was introduced.

The superior lamina of C2, ascending facet of C2, and inferior lamina were identified, followed by a C2 hemilaminectomy from cranial to caudal until reaching the superior border of the lamina. The hemilaminectomy was completed from medial to lateral up to the facet joint.

**Fig. 2:**
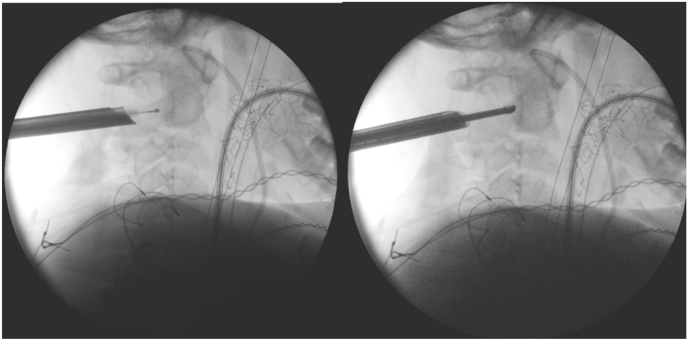
Intraoperative fluoroscopic images during endoscopic tumor resection. Left: radiofrequency ablation of the tumor bed. Right: curettage of the lesion bed.

The nidus was macroscopically identified. Intralesional curettage and biopsy sampling (multiple fragments) were then performed, achieving extensive resection until the cortical bone of the posterior border of the C2 vertebral body was visualized and palpated. This was complemented with radiofrequency ablation (RIWO®, up to 40 °C), confirming the absence of neuromonitoring changes (Fig. 2).

## 4 Results

Operative time was 3 hours with minimal blood loss.

In the immediate postoperative period, the patient evolved favorably, without neurological deficits and with significant relief of preoperative symptoms.

**Fig. 3:**
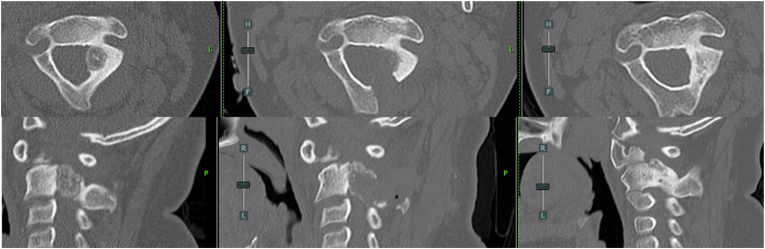
Comparative CT images (left to right): preoperative, immediate postoperative, and 3-year postoperative.

Immediate postoperative CT demonstrated left hemilaminectomy and near-complete nidus resection (Fig. 3). Histopathological analysis confirmed the diagnosis of osteoid osteoma.

Postoperatively, the patient experienced immediate pain relief without neurological deficits. During follow-up, clinical outcomes remained excellent: VAS 0/10 at rest and VAS 3/10 during lateral rotation, with preservation of cervical range of motion.

CT performed at 3 years demonstrated complete resection, no evidence of recurrence, and bone remodeling at the surgical site (Fig. 3).

## 5 Discussion

Osteoid osteoma involving the upper cervical spine is rare, but it may cause severe and disabling pain, particularly in pediatric patients. When symptoms are refractory to medical treatment, surgical or image-guided procedures are indicated (Kaner et al., 2010).

In high cervical locations, treatment selection is especially challenging due to the proximity of neural and vascular structures and the potential impact on craniocervical stability. Minimally invasive techniques, including radiofrequency ablation and endoscopic resection, have been increasingly reported as alternatives to open surgery.

NSAIDs remain the first-line treatment for pain control; however, prolonged use may be associated with gastrointestinal or renal complications and must be evaluated individually (Alemdar et al., 2016). In refractory cases, surgical excision via intralesional curettage or wide en bloc resection is considered, with the latter being the treatment of choice due to reported effectiveness of 88–100% (Kamrani et al., 2007). Nevertheless, wide resection in the cervical spine may be complex due to nearby critical structures and the risk of postoperative instability.

Therefore, minimally invasive techniques have gained popularity. Percutaneous radiofrequency ablation (RF), first described by Rosenthal in 1992 (Yang et al., 2018), has shown favorable results and is currently considered the gold standard for osteoid osteoma treatment. Despite this, concerns remain regarding its use in the cervical spine due to neural proximity and the risk of thermal injury (Ameri et al., 2019). CT-guided cryoablation has recently gained attention due to its theoretical advantage of reduced neural injury risk and navigated execution.

Minimally invasive RF was also ruled out due to vascular injury risk. Consequently, an endoscopic approach was selected to allow direct visualization of the nidus and surrounding structures while minimizing soft tissue damage (Cerny et al., 2022).

In this patient, a conventional microsurgical posterior approach could have been technically feasible without mandatory occipitocervical fixation, as suggested in previous reports (Kulkarni et al., 2013; Moore and McLain, 2005). However, such approaches may still require greater soft tissue dissection and bone removal, particularly in high cervical lesions located near critical neural and vascular structures.

The patient demonstrated excellent functional outcomes in both the immediate postoperative period and at 3-year follow-up, with significant pain reduction and return to age-appropriate daily activities. Radiographically, complete excision and progressive bone consolidation were confirmed on follow-up CT scans at 1 and 3 years.

## 6 Conclusion

Posterior endoscopic resection represents a minimally invasive and effective alternative for selected cases of high cervical osteoid osteoma when surgical treatment is indicated.

This approach allows direct visualization of the nidus, adequate tumor removal, and preservation of surrounding structures, with excellent clinical and radiological outcomes at 3-year follow-up in the present case.

Although other minimally invasive and microsurgical techniques remain valid options, this case supports the role of endoscopic surgery as a safe and feasible strategy in carefully selected patients.

This case represents the longest follow-up reported to date of a high cervical osteoid osteoma treated using an endoscopic approach.

## Declaration of competing interest

The authors declare that they have no known competing financial interests or personal relationships that could have appeared to influence the work reported in this paper.
